# The recommended treatment protocol for low-intensity shockwave therapy based on the severity of erectile dysfunction

**DOI:** 10.1038/s41443-024-00959-7

**Published:** 2024-08-06

**Authors:** Nikolaos Pyrgidis, Dimitrios Kalyvianakis, Ioannis Mykoniatis, Dimitrios Hatzichristou

**Affiliations:** 1 Institute for the Study of Urological Diseases, Thessaloniki, Greece; 2 Department of Urology, University Hospital, LMU Munich, Munich, Germany; 3 Department of Urology, Aristotle University of Thessaloniki, Thessaloniki, Greece

**Keywords:** Health care, Signs and symptoms, Erectile dysfunction, Urinary tract

## To the Editor:

Existing randomized controlled trials and meta-analyses indicate that, compared to sham treatment, low-intensity shockwave therapy (LiST) improves vasculogenic erectile dysfunction (ED) by about 4 points based on the International Index of Erectile Function—Erectile Function Domain (IIEF-EF) [1]. Nevertheless, the available studies have merged patients with mild, moderate, and severe ED, although these subsets of patients present different degree of underlying pathophysiology for ED and require a completely different therapeutic approach. The latter has rendered the interpretation of the available studies problematic, as it is not possible to inform our patients adequately on the expected efficacy of LiST.

In an attempt to harmonize clinical outcomes on the efficacy and safety of LiST based on the different degrees of ED, we aimed to provide a recommended treatment protocol based on the severity of ED. Based on the previous notion, we performed three parallel double-blind, randomized, sham-controlled clinical trials. Their characteristics are presented in Table 1. Patients with mild ED (IIEF-EF of 17–25) were randomized to either 6 LiST sessions (twice/week for three weeks) combined with daily 5 mg tadalafil (for four weeks) or LiST (twice/week for three weeks) with placebo (for four weeks) [2]. Patients with moderate ED (IIEF-EF of 11–16) were randomized to either 12 LiST sessions (twice/week for six weeks) or sham therapy (twice/week for six weeks) [3]. Patients with severe ED (IIEF-EF of 1–10) were randomized to either 12 LiST sessions (three times/week for four weeks) combined with daily 5 mg tadalafil (for four weeks) or sham therapy (three times/week for four weeks) with daily 5 mg tadalafil (for four weeks) [4].

**Table 1:** The characteristics of the included studies.

Study	Population	Treatment protocol	Number of patients	Follow-up evaluations	Mean difference between groups in IIEF-EF (95% CI)	Mean difference between groups in SEP3—YES (%) (95% CI)
Mykoniatis [2]	Mild ED (IIEF-EF: 17–25)	Group A: 6 LiST sessions (twice/week for 3 weeks) + 5 mg tadalafil (for 4 weeks) Group B: 6 LiST sessions (twice/week for 3 weeks) + placebo (for 4 weeks)	Group A: 25 Group B: 25	1 month, 3 months, 6 months	Baseline–1 month: 0.8 (−0.2 to 1.9), *p* = 0.12 Baseline–3 months: 1 (0.1 to 1.9), *p* = 0.02 Baseline–6 months: 1.7 (0.8 to 2.7), *p* < 0.001	Baseline–1 month: 2.3 (−10.4 to 15), *p* = 0.71 Baseline–3 months: 8.9 (−3.1 to 18.9), *p* = 0.07 Baseline–6 months: 11.8 (2.1 to 21.5), *p* = 0.02
Kalyvianakis [3]	Moderate ED (IIEF-EF: 11–16)	Group A: 12 LiST sessions (twice/week for 6 weeks) Group B: 12 sham sessions (twice/week for 6 weeks)	Group A: 35 Group B: 35	1 month, 3 months	Baseline–1 month: 3.9 (2.7 to 5.2), *p* < 0.001 Baseline–3 months: 4.4 (3.4 to 5.4), *p* < 0.001	Baseline–1 month: 19 (11 to 27), *p* < 0.001 Baseline–3 months: 23 (14 to 32), *p* < 0.001
Kalyvianakis [4]	Severe ED (IIEF-EF: 1–10)	Group A: 12 LiST sessions (three times/week for 4 weeks) + 5 mg tadalafil (for 4 weeks) Group B: 12 sham sessions (three times/week for 4 weeks) + 5 mg tadalafil (for 4 weeks)	Group A: 34 Group B: 17	1 month, 3 months	Baseline–1 month: 2.9 (2 to 3.9), *p* < 0.001 Baseline–3 months: 3.1 (2.1 to 4), *p* < 0.001	Baseline–1 month: 8.3 (−0.6 to 17), *p* = 0.07 Baseline–3 months: 7.1 (−2.2 to 16), *p* = 0.1

*CI* confidence interval, *ED* erectile dysfunction, *IIEF-EF* International Index of Erectile Function-Erectile Function, *LiST* low-intensity shockwave therapy, *SEP* sexual encounter profile.

In all studies, similar selection criteria, technique and treatment protocol were applied. In particular, the ARIES 2^TM^ LiST generator and the Smart Focus probe provided by Dornier MedTech GmbH, Wessling, Germany were used. During each session, a total of 5,000 impulses were applied to the penis (2000 impulses to the corpus, 2000 to the crura, and 1,000 to the penile base) based on the recommended protocol developed and published previously [5]. We applied an energy flux density of 0.096 mJ/mm^2^ with 5 Hz frequency (level 7 at the ARIES 2^TM^ device). The primary outcome in all studies was the effect of the recommended LiST treatment protocol after three months from treatment.

**Fig. 1:**
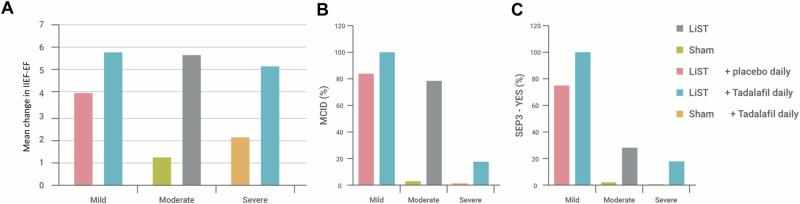
Efficacy of LiST at three months. The efficacy of LiST at three months after completion of the treatment protocol as a monotherapy or combination therapy in patients with different levels of erectile dysfunction based on the mean change of IIEF-EF domain from baseline (**A**), the number of patients attaining Minimal Important Clinical Difference (MCID) (**B**), and the proportion of “yes” responses to question 3 of the SEP diaries (**C**). IIEF-EF International Index of Erectile Function-Erectile Function, LiST low-intensity shockwave therapy, MCID minimal clinically important difference, SEP sexual encounter profile.

In patients with mild ED, the IIEF-EF, and the proportion of “yes” responses to question 3 of the Sexual Encounter Profile (SEP) diaries improved statistically significantly both in the LiST + tadalafil and in the LiST + placebo group at three months after completion of the treatment protocol. Importantly, combination therapy with LiST + tadalafil led to a statistically significant improvement of erectile function at three months post-treatment compared to LiST monotherapy. In patients with moderate ED, the IIEF-EF, the number of patients attaining minimally clinical important difference (MCID) and the proportion of “yes” responses to question 3 of the SEP diaries improved statistically significantly at the three-month evaluation after LiST monotherapy versus sham therapy. Similarly, in patients with severe ED, the IIEF-EF and the number of patients attaining MCID improved statistically significantly after LiST + tadalafil versus sham therapy + tadalafil at the three-month evaluation. In all studies, the LiST was well-tolerated with few, minor adverse effects. The efficacy of the recommended treatment protocol for LiST based on the severity of ED from our study group at the three-month evaluation is illustrated in Fig. 1.

The collective insights from these three double-blind sham-controlled randomized clinical trials emphasize the beneficial role of LiST both as a monotherapy and as a combination treatment modality in patients with different degrees of ED. From a clinical perspective, our studies suggest that adding daily low-dose tadalafil could substantially improve the efficacy of LiST, offering a robust alternative to traditional monotherapy approaches. In patients with moderate ED, LiST as a standalone therapy seems to be a safe and effective approach, especially in patients wishing to try another treatment modality apart from phosphodiesterase type 5 (PDE5) inhibitors. In patients with severe ED, our findings indicate that LiST combined with tadalafil may be an acceptable treatment option in those who are not willing to undergo more invasive therapies such as intracavernosal injections. In patients with severe ED, it seems that 20% of all patients benefit from 12 LiST sessions. The possibility to repeat LiST remains an option to be explored.

Despite our encouraging findings, all studies underline the necessity for larger, multicentric trials with diverse patient populations and longer follow-up periods. Moreover, a deeper insight into the synergic mechanism of LiST and tadalafil is also mandatory. Overall, these studies highlight the efficacy and safety of LiST both as a monotherapy and as a combination treatment modality with daily, low-dose tadalafil. In conclusion, six sessions of LiST combined with daily 5 mg tadalafil maximize efficacy in patients with mild vasculogenic ED. Accordingly, twelve sessions of LiST in regular PDE5 inhibitor users with moderate vasculogenic ED are an effective alternative treatment modality, and twelve sessions of LiST combined with daily 5 mg tadalafil may prove beneficial in partial responders to PDE5 inhibitors with severe vasculogenic ED who do not wish to receive more invasive treatments.

## Data Availability

The data supporting this study’s findings are available from the corresponding author upon reasonable request.
